# Effect of Vibration on Acute and Chronic Back Pain After Spinal Anesthesia: A Randomized Clinical Trial

**DOI:** 10.5812/aapm-143528

**Published:** 2024-03-11

**Authors:** Shervin Shahinpour, Fatemeh Refahi, Nader Ali Nazemian

**Affiliations:** 1 Department of Anesthesiology, School of Medicine, Tehran University of Medical Sciences, Tehran, Iran; 2 School of Medicine, Tehran University of Medical Sciences, Tehran, Iran

**Keywords:** Spinal Anesthesia, Back Pain, Vibration

## Abstract

**Background:**

Post-spinal anesthesia back pain often initiates with needle insertion and may persist for months, particularly among young women following cesarean section. Mechanical vibration has been proposed as an effective method to alleviate this pain.

**Objectives:**

The study aimed to evaluate the impact of vibration on reducing pain experienced during needle insertion, as well as its effects one week and one-month post-puncture.

**Methods:**

This randomized clinical trial enrolled patients undergoing spinal anesthesia for various surgical procedures. Patients were randomly assigned to either receive routine spinal anesthesia or spinal anesthesia combined with vibration. Demographic data were collected, and pain levels during needle insertion and back pain were assessed using a visual analog scale (VAS).

**Results:**

A total of 64 patients were included in the study. There were no significant differences between the two groups in terms of the number of attempts required for needle insertion (P = 0.341), the predominant anatomical level, or the needle approach (midline or paramedian). Ultimately, pain experienced during needle insertion, back pain after one week, and back pain after one month did not differ significantly between the two groups (P = 0.562, P = 0.14, and P = 0.267, respectively).

**Conclusions:**

The results of the present study showed that vibration at the site of needle insertion during spinal anesthesia had no effect on acute and chronic back pain on subsequent follow-up due to spinal anesthesia.

## 1. Background

Spinal anesthesia is routinely utilized in various lower abdominal surgeries, including cesarean section, herniorrhaphy, mesh implantation, jejunostomy, ileostomy, and gastrostomy (1-3). It has become an integral component of modern anesthesia due to its established efficacy, predictability, high patient satisfaction, and low complication rate. Technological advancements, such as improvements in needle diameter and design, have significantly reduced complications such as post-dural puncture headaches (PDPH) (4).

PDPB affects approximately 2 to 29% of patients following spinal anesthesia. It manifests as localized continuous pain at the site of spinal puncture without radicular pain (5), with reported incidence rates ranging from 2 to 29% (6, 7). Proposed mechanisms of PDPB include overstretching of spinal ligaments and local tissue trauma (5).

PDPB can lead to various adverse effects, delaying discharge from post-anesthetic care and hospitalization (8, 9). Additionally, patients often experience anxiety and distress during spinal anesthesia, with needle insertion pain being a significant concern. Moreover, pain during the procedure can result in involuntary patient movements, potentially leading to errors by anesthesiologists. Therefore, effective pain management during needle insertion is essential (9).

Some patients may continue to experience chronic back pain for months after spinal anesthesia. Based on the preemptive analgesia theory, mitigating acute needle insertion pain may help reduce the incidence of chronic back pain post-spinal anesthesia. Mechanical vibration has been shown to effectively alleviate acute and chronic pain, including needle pain, in both pediatric and adult populations. According to the gate control theory, mechanical vibration is expected to complement conventional pain control strategies by raising the pain threshold in the lumbar region (10, 11). While some studies have explored the effects of vibration on pain during cosmetic injections, its impact on lower back pain following spinal anesthesia remains unexamined. 

## 2. Objectives

This study aimed to investigate the efficacy of vibration in reducing acute pain and involuntary movements during spinal anesthesia, as well as its potential to prevent chronic back pain that may develop in the subsequent week or month.

## 3. Methods

### 3.1. Subjects

This study was conducted as a parallel clinical randomized trial (IRCT20220107053657N1) approved by the Ethics Committee of Tehran University of Medical Sciences and adhered to the principles of the Helsinki Declaration. Patients who agreed to participate provided informed consent (IR.TUMS.MEDICINE.REC.1400.155). Considering an anticipated 29% incidence of back pain after spinal anesthesia in the control group and aiming for a reduction in post-dural puncture back pain (PDPB) to 3% (primary outcome), a minimum sample size of 30 patients per group was calculated (with an alpha error of 5% and a study power of 80%). The study population comprised patients undergoing spinal anesthesia for surgical procedures at the Imam Khomeini Hospital complex from January to June 2022. 

The inclusion criteria were as follows: 

(1) Patients aged 20 to 70 years

(2) American Society of Anesthesiologists (ASA) scores of 1 to 3

(3) Patients undergoing spinal anesthesia for herniorrhaphy, mesh implantation, jejunostomy, or ileostomy surgery.

Exclusion criteria included patients who received general anesthesia due to the cancellation of spinal anesthesia, as well as those who required narcotics or ketamine to continue surgery due to incomplete spinal anesthesia. Patients with risk factors for PDPB, such as immobilization exceeding 2.5 hours, a history of back pain, or a body mass index (BMI) over 32 kg/m^2^, were also excluded.

A computer-generated randomization table (using Microsoft Excel) was utilized to allocate patients into two groups: Control and intervention. Thirty-two patients were assigned to the control group, where spinal anesthesia was administered without a vibrator, while 32 patients were assigned to the intervention group, where a vibrator was placed near the needle insertion site. Demographic data, including age, sex, and BMI, were collected from patients. Pain and involuntary movement during needle insertion, as well as postoperative back pain, were assessed using the visual analog scale (VAS) criteria. Back pain was further monitored via telephone follow-up one week and one month after surgery. Data collection and analysis were performed in a blinded manner.

### 3.2. Procedure

Before administering spinal anesthesia, patients underwent monitoring, and intravenous (IV) access was established. Positioned in a sitting posture with either a midline or paramedian approach at the L4 - L5 level, the targeted area was initially sterilized. A sterile cover housing the vibrating device was positioned approximately 1 to 2 cm away from the injection site. The vibration stimulus, set at a frequency of 20 - 30 Hz, commenced ten to twenty seconds before spinal anesthesia initiation and persisted until needle withdrawal from the skin. The vibrating device and frequency remained consistent across all participants. Upon spinal needle insertion, the vibrator was displaced 1.5 cm away, and the anesthesiologist administered bupivacaine 0.5% at a dosage of 10 - 15 mg, depending on the surgical site. Pain during needle insertion and subsequent back pain were monitored by a blinded colleague regarding patient groups.

### 3.3. Statistical Analysis

Data analysis was performed using the Statistical Package for Social Sciences 20 (SPSS) software (SPSS Inc., Chicago, IL, USA). Mean and standard deviation were utilized for quantitative data representation, while frequency was employed for qualitative data. A significance level below 5% was considered statistically significant. Changes in outcome variables over time were assessed using repeated measures analysis of variance (ANOVA). The chi-square test compared categorical data, while the independent-sample *t*-test analyzed normally distributed continuous variables, and its non-parametric equivalent (Mann-Whitney test) was employed for continuous variables with non-normal distribution. The correlation between observations within the same subject was 0.3, and the alpha level was set at 0.05.

## 4. Results

In this study, 32 patients were allocated to the vibrator group, and 32 patients were assigned to the control group. There were no significant differences in age, gender, or other demographic variables between the two groups. The mean age in the vibrator and non-vibrator groups was 36.09 ± 4.06 and 37.9 ± 12.49, respectively, showing no significant difference (P = 0.6). In the vibrator group, 21 patients were female (67.74%), and 10 patients (32.25%) were male, while in the non-vibrator group, 9 were male (27.3%) and 24 (72.7%) were female, which did not differ significantly (P = 0.08). The mean BMI in the vibrator group was 32.9 ± 4.91 kg/m^2^, and in the control group, it was 29.18 ± 3.49 kg/m^2^ (P = 0.11) (Table 1). 

**Table 1.** Demographic Information of Patients ^a^

Variables	Vibrator (n = 32)	Control (n =3 2)	P-Value
**Age**	36.09 ± 4.06	37.9 ± 12.49	0.6
**Gender**			0.08
Male	11 (34.37)	10 (31.25)	
Female	21 (65.63)	22 (68.75)	
**Body Mass Index**	32.9 ± 4.91	29.18 ± 3.49	0.11

^a^ Values are expressed as mean ± standard deviation or No. (%).

**Figure 1.**
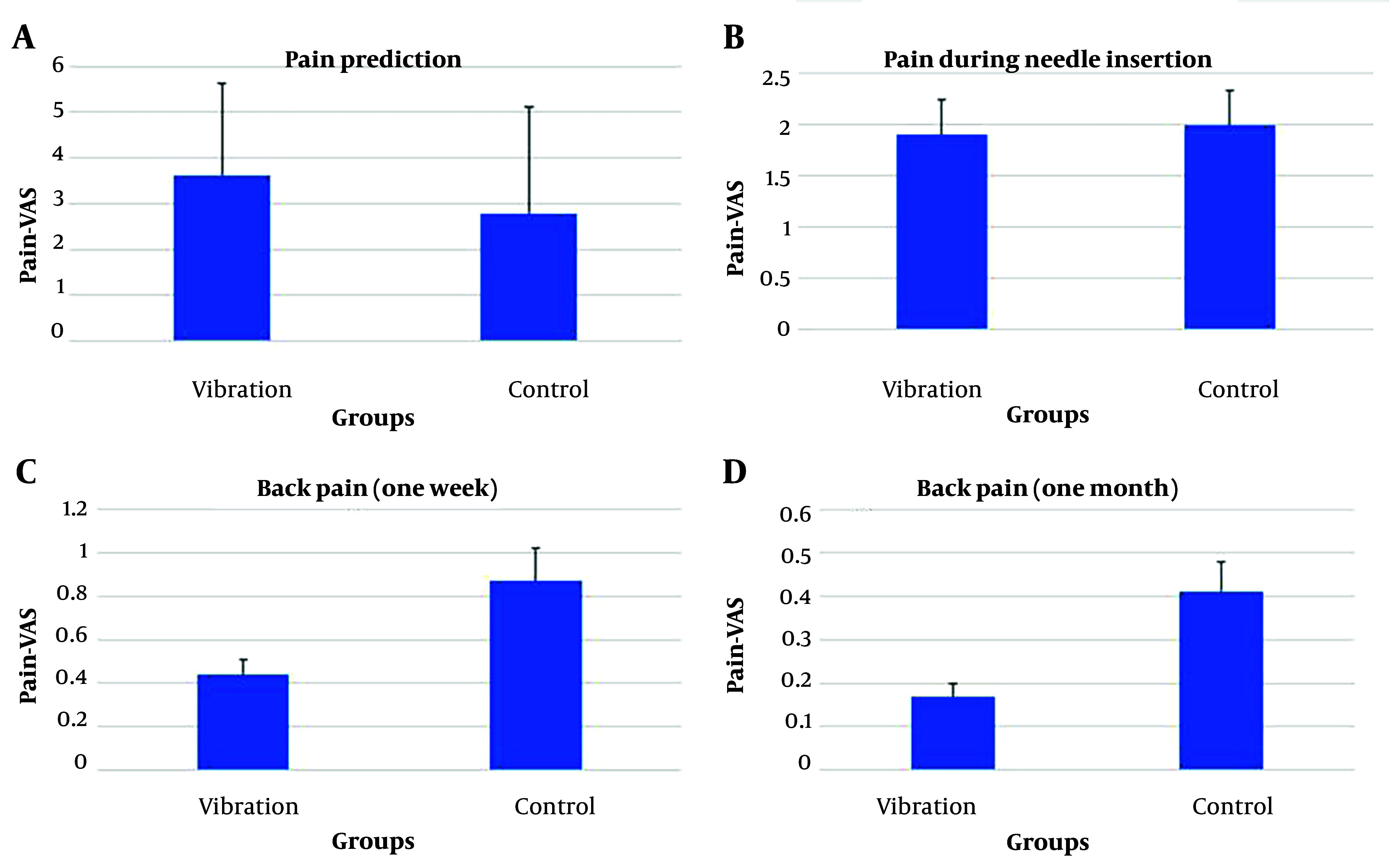
The severity of back pain one week and one month after surgery

Clinical data of the two groups were compared, as indicated in Table 2. There was no significant difference in the number of attempts to insert the needle between the two groups (P = 0.341). The predominant anatomical level in both groups was L4 - L5 (P = 0.761), and the needle approach (midline and paramedian) did not differ between the two groups (P = 0.347). Although the number of sudden movements was higher in the vibrator group, there was no statistically significant difference between the two groups (P = 0.087). Predictions of pain did not significantly differ between the two groups (P = 0.128). Furthermore, there was no difference between the two groups regarding pain during needle insertion (P = 0.562). Additionally, the severity of back pain did not significantly differ one week and one month after surgery (P = 0.14 and P = 0.267, respectively) (Figure 1). 

**Table 2.** Comparison of Clinical Information Between the Vibrator and Control Groups ^a^

Variables	Vibrator (n = 32)	Control (n = 32)	P-Value
**Attempt to insert the needle**	0.83 ± 0.15	0.42 ± 0.08	0.341
**Anatomic level**			0.761
L2 - L3	1	2	
L3 - L4	11	11	
L4 - L5	20	19	
**Approach of needle**			0.347
Midline	28	31	
Para median	4	1	
**Sudden movement when needling**			0.087
Yes	14	7	
No	18	25	
**Pain prediction**	3.61 ± 2.02	2.78 ± 2.33	0.128
**Pain during needle insertion**	1.9 ± 0.34	1.99 ± 0.34	0.562
**Back pain (after a week)**	0.44 ± 0.07	0.87 ± 0.15	0.14
**Back pain (after a month)**	0.17 ± 0.03	0.41 ± 0.07	0.267

^a^ Values are expressed as mean ± standard deviation or No.

## 5. Discussion

PDPB has various risk factors, including lithotomy position, immobilization for more than 2.5 hours, a history of back pain, a BMI exceeding 32 kg/m^2^, and multiple needle punctures (2, 12-16). Patients with these risk factors were excluded from this study.

Considering the impact of vibration observed in previous studies, our research aimed to investigate the effect of vibration near the site of needle insertion on reducing acute pain and involuntary movement during spinal anesthesia, as well as potential chronic back pain that may arise after one week or one month. Literature suggests that employing gentle injection techniques and thin needles can minimize pain during injections. Additionally, topical application of ice packs, cooling sprays, and anesthetic creams has proven beneficial (17-20).

The mechanism of vibrational anesthesia is largely associated with the "gate control theory," proposed by Melzac and Wall in 1965 as cited by Cohen et al.. According to this theory, pain sensation is regulated by intrinsic neurons and controls originating from the brain, with gating synapses controlling the amount of pain signals reaching the brain. Activation of vibratory fibers (A-beta) is believed to reduce pain signals transmitted by pain fibers (A-delta and C fibers) (21).

However, pain transmission is likely more complex, as the gate control theory does not fully explain all types of pain, such as phantom limb syndrome (22). Studies have indicated that vibrational stimuli can stimulate afferents in Pacini cells, receptors on the skin, periodontium, muscle spindles, and tendon organs (23).

Vibration can serve as a safe method for inducing local anesthesia. Although occupational studies suggest that chronic exposure to intense whole-body vibration may increase the risk of spinal degeneration (24, 25), short-term exposure to local vibrations is not associated with significant temporary or permanent side effects, to our knowledge. Nevertheless, prolonged vibration exposure through the hands may lead to vascular or neurological changes in the upper extremities (26).

The results reported in various studies regarding the effect of vibration on pain have yielded conflicting findings. Studies conducted in dentistry have indicated that vibration decreases the pain experienced by patients, contrary to the results of our study. In our research, pain scores during needle insertion and back pain one week and one month later did not differ between the two groups. Pujari et al. (26) concluded that vibration techniques effectively reduce pain and anxiety in patients undergoing anesthesia.

The same results by Pasterczyk-Szczurek et al. (27) showed that adult patients in the vibration group experienced less pain during anesthesia compared to the control group. They assessed pain levels using the VAS and the McGill Pain Questionnaire. Additionally, they utilized extraoral vibrations, which may mitigate the effect of vibrations applied through the pain control gate mechanism due to the distance between the injection site and the device (27). Furthermore, Sharma et al. (28) demonstrated a significant reduction in injection pain and discomfort after using their dental vibe injection system in adult volunteers.

In another study investigating the safety and effectiveness of vibration in reducing pain caused by BTX-A injection, it was shown that vibration effectively reduces pain and may be applicable in other cosmetic procedures (28-30). However, Roeber et al. (31) found no difference in the level of injection pain between conventional injection and injection with vibration assistance.

One limitation of studies assessing pain levels using different injection systems, including ours, is the inability to measure pain levels objectively. Mental techniques, such as marking on the VAS or selecting the corresponding facial image indicating the level of pain, are commonly employed. In our study, VAS measurements were utilized to compare pain levels between groups, consistent with previous studies involving pediatric and adult patients (32). Since pain perception is multifactorial, as physicians, we must acknowledge the patient's description of pain levels. Therefore, despite the subjective nature of these assessment methods, their current use is appropriate for evaluating pain.

### 5.1. Conclusions

In conclusion, our study found that vibration at the site of needle insertion during spinal anesthesia did not affect pain during needle insertion, nor did it significantly influence back pain one week and one month later. Given that our study was conducted in a referral center by experienced individuals who successfully accessed the subarachnoid space with a single attempt using a small 25-gauge spinal needle, the pain-reducing effect of the vibrator was not observed significantly. Hence, future studies should consider conducting similar research on inexperienced anesthesiology residents.

## Data Availability

The dataset presented in the study is available on request from the corresponding author after publication. The data are not publicly available due to privacy of research participants.

## Clinical Trial Registration Code:

IRCT20220107053657N1.
